# Relationships of work characteristics to job satisfaction, turnover intention, and burnout among doctors in the district public-private mixed health system of Bangladesh

**DOI:** 10.1186/s12913-017-2369-y

**Published:** 2017-06-20

**Authors:** Ashim Roy, Trudy van der Weijden, Nanne de Vries

**Affiliations:** 1 Department of Health Promotion, School CAPHRI, Faculty of Health, Medicine and Life Sciences, Maastricht University, Post Box: 616, Post code: 6200, MD Maastricht, the Netherlands; 2 Department of Community Health, Graduate Health Project, Joypurhat, 5900 Bangladesh; 3 Department of Family Medicine, School CAPHRI, Faculty of Health, Medicine and Life Sciences, Maastricht University, Maastricht, the Netherlands

**Keywords:** Work design, Job satisfaction, Turnover intention, Burnout, Health system, Bangladesh

## Abstract

**Background:**

Work design integrates work characteristics having organizational, social and job components which influence employees’ welfare and also organizational goals. We investigated the effects of work characteristics and other predictors to job satisfaction, turnover intention, and burnout in doctors of the public primary, public secondary and private facilities of the district health system of Bangladesh.

**Methods:**

A quantitative study using a self-administered questionnaire containing mostly structured items was conducted among the public and private doctors with a sample size of 384 from 29 out of a total 64 districts of Bangladesh during October and November 2015. All variables including work characteristics and outcomes of interest were based on literature and measured on 5-point Likert scale. Multivariate analysis of variance, bivariate correlation, and multiple regression were the models operated through SPSS version-21.

**Results:**

A total of 354 doctors responded. No significant differences were found between public primary and secondary level doctors on combined work characteristics and outcomes variables, which however differed significantly between the public and private doctors. Organizational support was the strongest predictor adversely affecting job satisfaction, turnover intention and burnout of both the public and private doctors; private doctors’ experienced more support. The effects of health-professional politics on the public doctors were alarming.

**Conclusion:**

Work design of the Bangladesh’s health system is in need of ample development. Doing so, improvement in organizational supports is crucial; however, other work characteristics components are also important for enhancing doctors’ welfare and health system productivity.

**Electronic supplementary material:**

The online version of this article (doi:10.1186/s12913-017-2369-y) contains supplementary material, which is available to authorized users.

## Background

In many developing countries, inefficient work design and ineffective human resource management (HRM) are among the key barriers to attain the universal goals of the health system i.e. optimal and equitable response to people’s needs for health and economic protection [1, 2]. Improvement in work design as well as HRM policy is essential for attaining the key national health policy goal of universal access to primary health care [3].

A raft of empirical evidence has been generated over decades suggesting that strategic work design is essential to improve job holders’ attitudinal, behavioural and emotional states to achieve individual as well as organizational interests and goals [4, 5]. Conceptually, work design integrates ‘work characteristics’ such as organizational, social and job components having the potential to determine individual and organizational interests and outcomes [6]. Notably, job-holders’ attitude (e.g. job satisfaction), behaviour (e.g. turnover intention) and well-being (e.g. burnout) states are commonly-studied work design outcomes which are under the complex influence of the wider organizational environment [4, 6–8].

Job satisfaction of health professionals (e.g. doctors and non-doctor staff) is essential since their dissatisfaction is linked to poor quality of care and poor health outcomes. These are conducive to patients’ dissatisfaction; hence, suboptimal productivity of the healthcare delivery system as well [9, 10]. Again, poor job satisfaction is associated with turnover of health care staff; oppositely, enhancement of job satisfaction and working environment is potentially effective to improve retention and quality of care [11] . Empirically, structural, managerial and staff welfare factors (i.e. organizational factors), social factors and work in it-self (i.e. job characteristics) are the key attributes influence job satisfaction and turnover or retention of health professionals [10, 12]. Further, a study at Ghana showed that strengthening of human resource management skills of the district level health managers are quite important for optimizing health professionals’ motivation, job satisfaction and retention [13].

### Human resource management in health system: Bangladesh scenario

In Bangladesh, health care services are delivered through a public-private mixed provision. While the public health sector is tax- and donor-based, the private health sector is market-based. Whereas the public health system consists of a country-wide network of primary, secondary and tertiary level facilities, the private health sector comprises with mostly urban-based secondary and tertiary level facilities. The public primary and both public and private secondary level health facilities constitute a district health system. The district health care system is the main source of health care to the rural residents who occupy nearly 72% of over 151 million country population [14]. The tertiary level health facilities are the graduate and post-graduate training institutions. In Bangladesh, to be a registered graduate doctor (i.e. Bachelor of Medicine and Bachelor of Surgery (MBBS)), a five-year successful institutional training followed by 1 year internship is mandatory. To be a specialist doctor, there are diploma, fellowship and doctorate degrees which require additional 3–5 years post-graduate trainings. All graduate (i.e. MBBS) doctors are General Physicians (GPs). In the public sector, whereas the senior GPs usually are promoted to administrator positions, the post-graduate doctors are upheld to consultant posts (i.e. specialists) in primary and secondary level facilities or academic positions (i.e. assistant / associate / full professors) in tertiary level facilities. Both the public primary and secondary level facilities have defined posts for GPs and consultant doctors. In the public sector, all doctors initially employed as Medical Officer. Both a GP and a post-graduate doctor could be Medical Officers and a post-graduate Medical Officer initially is promoted to a Junior Consultant after fulfilling maturity criteria (personal experience).

Bangladesh is one of the 57 countries of the world with a serious shortage of human resource in health care with an approximate doctor-population ratio of 3:10,000. Currently to fulfil the WHO standard at least 90,000 more doctors would be needed. In the public-private mixed health system of Bangladesh, nearly 62% of all doctors are working in the private and the rest in the public sector. The majority of the public doctors are involved in the private sector as dual-practitioners. The private health facilities are mostly urbanized. Also an urban concentration of public doctors is a known problem that renders the rural doctors’ posts vacant with consequent high workloads and rural people’s poor access to quality health care [3].

Within limitations the health system of Bangladesh has recognizable achievements; for example, reduction in the infant mortality rate and maternal mortality ratio [3]. Doctors’ are among the key internal clients of the health system and their role in achieving organizational goals is quite important; hence, their fit and satisfaction with the organizational environment are also essential [1, 15]. However, some studies reported that doctors’ behavioural and technological deficiencies are mainly linked to patients’ dissatisfaction in the health care system of Bangladesh (e.g. [16, 17]). Notably, most of those studies were based on patients’ opinions only without taking into account doctors’ opinions and the wider organizational culture and context which potentially influence their attitude, behaviour and well-being. Since doctors of the district health system are the key service providers to the majority of the rural people, their views of the impacts of the wider organizational environment deserve investigation. Thus, we investigated whether or not the work characteristics (i.e. organizational factors, social factors and job characteristics) are associated with any problems in job satisfaction, turnover intention, and burnout in doctors of the district health system of Bangladesh.

Therefore, this study aimed: (1) to identify the relationships of key attributes of work characteristics to job satisfaction, turnover intention, and burnout in doctors of the district public primary, public secondary, and private health care facilities, and whether or not the relations differ between the doctors in those categories of health facility, and (2) to determine the key predictors of work characteristics influencing outcomes of interest in them.

## Methods

### Study design, population and settings

A quantitative study was conducted. Doctors of the public primary (i.e. upazilla or sub-district), public secondary (i.e. district), and private health care facilities were the target population. Doctors in tertiary level facilities were not included. General physicians (GPs), specialist doctors of diverse disciplines including dentists who were deployed in public and private facilities of the district health care system were targeted. Doctors were included if they had at least 2 years of job experience in an organized private or public facility. Twenty nine districts out of a total 64 districts of all seven divisions of the country were selected. The following two issues were taken into account for selecting districts: wider geographic representation, and availability of potential volunteer/s for collecting data from doctors. A sample size of 384 was calculated based on an infinite population size, a 95% confidence interval and since there was no data available on the issues, we assumed ±5% precision, and 50% degree of variability in outcomes. Equal numbers of doctors (*n* = 128) were to be sampled from each of the private sector, and public primary and secondary levels.

### Conceptual model

**Fig. 1:**
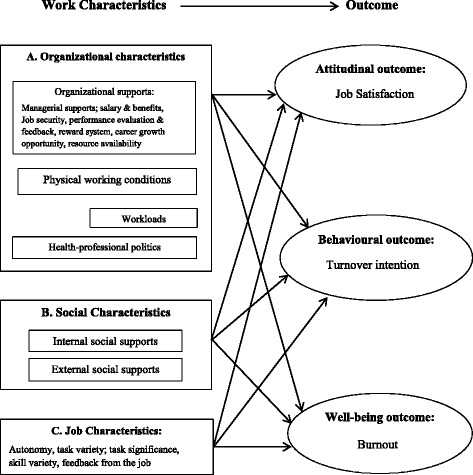
Conceptual model shows domains and attributes of work characteristics and outcomes of work design for the public and private doctors of the district health system of Bangladesh (adapted)

The Fig. 1 represents the conceptual model of this study. The key domains and attributes of work design are based on literatures [4, 6–8], except *health-professional politics*. The Bangladesh Medical Association (BMA) is the state-affiliated organ of doctors. The democratic leadership of the BMA usually follows national main stream politics. There are notions that doctors’ transfer, posting and promotion often are based on their political influence resulting in individual benefits or deprivation. During the design of the study tools, senior doctors suggested to assess doctors’ views regarding the impacts of ‘health-professional politics’.

### Data collection tools and techniques

A self-administered questionnaire with mostly closed items was developed by using existing work characteristics inventories [18, 19] and by discussions with 12 senior doctors (public *n* = 7; private *n* = 5); (please see the ‘Additional file 1’ for the questionnaire that has been uploaded as supplementary material). A few qualitative open-ended items were also included; for example, doctors’ daily and weekly working hours, and average number of patients they serve daily in the public and/or private sector. Section 1 contained questions about personal and professional background. Section 2 consisted of three work characteristics domains (Fig. 1). Content validity of the scales on influence of ‘health-professional politics’ was ensured through the senior doctors’ full consensus. Section 3 contained questions on outcomes: job satisfaction (using the global satisfaction scale of Lichtenstein [20]), turnover intention (using the scale described by Houkes et al. [8]), and burnout (using the inventory of West et al. [21]). All quantitative variables were measured with 5-point Likert scales (1 = strongly disagree, 2 = disagree, 3 = neither agree nor disagree, 4 = agree, and 5 = strongly agree), except ‘burnout’ which was measured on 7-point Likert scale. Table 1 presents the composition and internal consistency coefficients of the scales.

**Table 1:** Cronbach’s α statistics of different variables including number of items

Domains	Cronbach’s α	Items
Variables		
Organizational characteristics	.77	18
Organizational supports	.83	8
Physical working condition	.82	5
Workloads	.79	2
Health-professional politics	.81	3
Social characteristics	.72	4
Internal social supports	.68	2
External social supports	.65	2
Job Characteristics	.72	9
Autonomy	.68	2
Task variety	.66	4
Task significance	-	1
Feedback from job	-	1
Skill variety	-	1
Outcomes		
Satisfaction	.73	3
Turnover intention	-	1
Burnout	.70	3

The Principal Investigator contacted his colleagues and friends, mostly doctors, in all regions of the country who volunteered to coordinate data collection. The study objectives, inclusion criteria and explanations on the questionnaire were presented to them face-to-face and electronically. It was not possible to use the existing lists of doctors at the public primary and secondary level facilities as the sampling frame and to apply random sampling method because of the following reasons: a considerable number of doctors were absent in facilities where they were primarily enlisted, and enrollment in post-graduate training in tertiary level facilities. Moreover, public doctors were also enlisted in the private facilities as duty doctors for working out of public office-time; thus, use of random sampling might cause duplication of public doctors. For these reasons, a convenience sampling method was applied. Questionnaires were distributed among the volunteers at the selected districts via postal mails. The volunteers then distributed questionnaires to the pre-selected doctors who fulfilled the inclusion criteria and expressed interest of participating in the study. Written informed consent was given by all respondents. For confidentiality reason, all respondents sealed the questionnaires in envelopes prior to returning them to the volunteers who finally sent them to the Principal Investigator via postal mails. Data was collected during October and November 2015.

### Statistical analysis

SPSS (version 21) was used for processing and analyzing the data. Internal consistency of the scales was assessed by Cronbach’s α statistic as recommended by Nunnally [22] and was acceptable (Table 1). Linearity and normality were confirmed by partial plot and p-p plots respectively. Presence or absence of outliers was determined using Cook’s distance (cut-off point <1) and that of multicollinearity by variance inflation factors (cut-off point <10) as well as by tolerance factors (cut-off point >0.1) as described by Tabachnick & Fidell [23]. No serious violations of assumptions were noted.

Means and standard deviations were inspected. One-way multivariate analysis of variance was performed to test any significant differences in work characteristics and outcomes between the doctors of the public primary and public secondary level facilities, and between all public and private doctors. Bivariate correlation analyses were conducted to detect the direction and strength of relations between work characteristics and outcomes. Multiple regression (forward method) was used to identify the key predictors of the outcomes of interest. Ninty 5 % bias corrected and accelerated bootstrap confidence intervals (BCa CIs) were used to assess significance of correlation. An online programme (http://vassarstats.net/rdiff.html) was used to test any significant differences in the corresponding correlation coefficients between the public and private sector using Fisher’s r-to-z-transformation. A *p*-value <.05 was regarded as significant. Missing data were treated by using the ‘listwise’ option of SPSS.

## Results

### Background information of the respondents

A total of 354 doctors (public primary *n* = 121, public secondary *n* = 117, and private *n* = 116) from 29 districts in all 7 divisions of Bangladesh responded. The response rate was over 92%. The overall ratio of male to female doctors was nearly 70:30 which matches with the national ratio of 72:28 [3]. Respondents’ mean age was 39.2 (± 8.3) years with an average working experience of 10.8 (± 7.4) years. Table 2 shows background information of the respondents.

**Table 2:** Background information of the respondents

Variables Categories	Public PHC (*n* = 121) Number (%)	Public SHC (*n* = 117) Number (%)	Public total (*n* = 238) Number (%)	Private (*n* = 116) Number (%)
Job position				
Medical Officer	87 (72)	73 (62)	160 (67)	94 (81)
Junior Consultant	29 (24)	27 (23)	55 (23)	15 (13)
Senior Consultant	0 (0)	10 (8)	10 (4)	5 (4)
RMO	5 (4)	5 (4)	9 (4)	2 (2)
EMO	0 (0)	4 (3)	4 (2)	0 (0)
Qualification/specialization				
Graduate- MBBS/GP	82 (68)	58 (49)	140 (59)	80 (69)
Postgraduate/Specialist	39 (32)	57 (49)	96 (40)	34 (29)
Dentists	-	2 (2)	2 (1)	2 (2)
Dual practice				
Yes	96 (79)	89 (76)	185 (78)	69 (59)
No	25 (21)	28 (24)	53 (22)	47 (41)
Geographic distribution by division (No. of districts)				
Dhaka (5)	18 (15)	11 (9.5)	29 (12)	24 (21)
Chittagong (6)	12 (10)	25 (21)	37 (16)	19 (16)
Rajshahi (6)	26 (22)	25 (21)	51 (21)	23 (20)
Khulna (2)	19 (16)	10 (9)	29 (12)	19 (16)
Sylhet (2)	9 (7)	11 (9.5)	20 (8)	7 (6)
Barisal (2)	16 (13)	5 (4)	21 (9)	7 (6)
Rangpur (6)	21 (17)	30 (26)	51 (21)	17 (15)

*Notes*: *PH* Primary Health Care, *SHC* Secondary Health Care, *EMO* Emergency Medical Officer, *RMO* Residential Medical Officer, *MBBS* Bachelor of Medicine and Bachelor of Surgery, *GP* General Physician

### Descriptive statistics with multivariate analysis of variance

Means and standard deviations of variables of work characteristics and primary outcome domains in the public and private sector are presented in Table 3. These are based on significant differences between the public and private sector as identified through multivariate analysis of variance (MANOVA).

**Table 3:** Descriptive statistics of work characteristics and outcomes variables in the public and private doctors

Domains	Public (*n* = 238)	Private (*n* = 116)	*p*-values
Variables	Mean (SD)	Mean (SD)	
Organizational characteristics			
*Organizational supports	2.47 (± 0.65)	3.24 (± 0.77)	<.001
*Physical working condition	2.70 (± 0.79)	3.58 (± 0.78)	<.001
Workloads	3.60 (± 0.95)	3.50 (± 0.89)	.396
*Health-professional politics	3.74 (± 0.88)	3.05 (± 1.12)	<.001
*Grand mean OC	3.13 (± 0.43)	3.34 (± 0.53)	<.001
Social characteristics			
*Internal social supports	3.53 (± 0.87)	3.99 (± 0.79)	<.001
*External social supports	3.73 (± 0.75)	4.17 (± 0.66)	<.001
*Grand mean SC	3.63 (± 0.67)	4.08 (± 0.62)	<.001
Job Characteristics			
*Autonomy	3.34 (± 0.85)	3.76 (± 0.90)	<.001
*Task variety	3.29 (± 0.65)	3.66 (± 0.67)	<.001
Task significance	4.34 (± 0.81)	4.42 (± 0.63)	.350
*Feedback from job	3.78 (± 0.95)	4.15 (± 0.74)	<.001
Skill variety	4.13 (± 0.92)	4.12 (± 0.84)	.992
*Grand mean JC	3.57 (± 0.52)	3.87 (± 0.48)	<.001
Outcomes			
*Job satisfaction	2.95 (± 0.81)	3.24 (± 0.78)	.002
Turnover intention	3.06 (± 1.14)	3.19 (± 1.24)	.189
*Burnout	2.89 (± 1.24)	2.09 (± 1.03)	<.001

*Notes*: Asterisks (*) before the variables indicate that those variables are significantly different between the public and private sector. *OC* Organizational characteristics, *SC* Social characteristics, *JC* Job characteristics

MANOVA revealed no significant differences between doctors of the public primary and public secondary facilities on the combined work characteristics variables (*F*_7, 222_ = 1.55; *p* = .15; Wilks’ Lambda = .95; partial eta squired = .047), and outcomes of interest (*F*_3, 234_ = 4.67; *p* = .003; Wilks’ Lambda = .94; partial eta squired = .056). Separate analysis of work characteristics variables found that none of them were significantly different with a Bonferroni adjusted *p*-value of <.007. Although Wilks’ Lambda was significant for the combined outcome variables; in separate analysis only ‘burnout’ was found significant (*p* = .002) using a Bonferroni corrected *p*-value of <.017; however, the effect size was quite small (partial eta squared = .005). So, we treated public doctors as one group.

We found statistically significant differences between the public and private doctors on the combined work characteristics variables (*F*_7, 331_ = 21.48; *p* = <.001; Wilks’ Lambda = .69; partial eta squired = .312), and outcomes of interest (*F*_3, 346_ = 15.70; *p* = <.001; Wilks’ Lambda = .88; partial eta squired = .12). Separate analysis of work characteristics variables showed that all variables had statistically significant differences with a Bonferroni adjusted *p*-value of <.007 except for workloads; ratings by private doctors were more positive. Of the outcome variables all but turnover intention was found significantly different using a Bonferroni corrected *p*-value of <.017; again private doctors gave more positive judgements.

Among the variables of work characteristics and outcomes having significant differences between the public and private doctors, means of all but health-professional politics and burnout variables were greater in the private than in their public counterparts. In both sectors, combined organizational characteristics were evaluated less positively than social and job characteristics. Of all the variables of work characteristics, organizational supports were scored lowest in both the public and private sectors. Among the outcome variables, burnout in the private sector was scored lowest even notably lower than the neutral value (Table 3).

The reported daily official working hours in the private facilities varied from 8 to 10 h which is markedly higher than the usual 6.5 h in the public sector. However, because of also doing private practice, the calculated total working hours/week was marginally higher in public doctors than in their private counterparts, 60.3 (± 18.5) hours versus 57.7 (± 16.4) hours respectively. Of all respondents, approximately 58% worked 6 days/week (public 58%; private 64%), 41% worked 7 days/week (public 42%; private 35%) and only 1% of private doctors worked 5 days/week. Each public and private doctor treated on average respectively nearly 50 (±31) patients and 34 (±20) patients per day. In addition to their public sector patients, the dual-practitioners treated an average of 12 (± 11) patients daily in private practice.

### Correlation statistics

Overall, no significant differences in correlation coefficients between the public and private sector were found except for the relations between health-professional politics and job satisfaction. However, considering the organizational contexts, arithmetic differences in the magnitude of *r* values in the public and private sector seem more meaningful than statistical significance. Table 4 presents the correlation model statistics.

**Table 4:** Correlations between work characteristics and outcomes variables of work design in the public and private doctors including 95% BCa CI; and *p*-values

Work characteristics	Provider	Outcome variables		
		Satisfaction	Turnover intention	Burnout
		Pearson’s correlation coefficient - *r*; 95% BCa CI; *p*-value (*p*-values (two-tailed) of differences in transformed *r*-to-z between the two sectors)		
Organizational supports	Public	.370; [0.25, 0.48]; <.001	−.420; [−0.52, −0.31]; <.001	−.214; [−0.34, −0.06]; .001
	Private	.534; [0.35, 0.70]; <.001 (.07)	−.375; [−0.52, −0.21] < .001 (.64)	−.129; [−0.30, 0.04]; .17 (.44)
Physical working condition	Public	.335; [0.20, 0.45]; <.001	−.181; [−0.31, −0.06]; .005	−.083; [−0.21, −0.05]; .20
	Private	.338; [0.16, 0.51]; <.001 (.97)	- .308; [−0.49, −0.10]; .001 (.24)	−.184; [−0.34, −0.02]; .05 (.37)
Workload	Public	−.018; [−0.18, 0.17]; .77	.145; [−0.01, 0.27]; .03	.179; [0.07, 0.28]; .006
	Private	−.010; [−0.21, 0.20]; .92 (.97)	.014;. [−0.20, 0.21]; .88 (.25)	.060; [−0.15, 0.26]; .32 (.29)
Health- professional politics	Public	−.221; [−0.37, −0.06]; .001	.270; [0.11, 0.41]; <.001	.210; [0.10, 0.32]; .001
	Private	.077; [−0.12, 0.27]; .42 (.008)	.082; [−0.29, 0.13]; .39 (.08)	.005; [−0.15, 0.16]; .96 (.06)
Internal social support	Public	.224; [0.08, 0.36]; .001	−.085; [−0.22, 0.06]; .19	−.050; [−0.17, 0.09]; .44
	Private	.322; [0.12, 0.54]; .001 (.35)	−.250; [−0.40, −0.09]; .01 (.13)	−.222; [−0.36, −0.08]; .02 (.13)
External social support	Public	.295; [0.17, 0.40], <.001	−.008; [−0.14, 0.14]; .90	−.117; [−0.24, −0.002]; .07
	Private	.162; [−0.07, 0.39]; .09 (.22)	−.093; [−0.27, 0.09]; .33 (.46)	−.291; [−0.45, −0.14]; .002 (.11)
Job characteristics	Public	.359; [0.20, 0.51]; <.001	−.040; [−0.18, 0.09]; .538	- .149; [−0.26, −0.03]; .02
	Private	.196; [0.02, 0.36]; .04 (.12)	−.230; [−0.40, −0.06]; .016 (.08)	- .217; [−0.38, −0.06]; .02 (.54)
Satisfaction (a secondary outcome)	Public	-	−.427; [−0.55, 0.27]; <.001	−.088; [−0.21, 0.04]; .17
	Private	- -	−.358; [−0.52, −0.12]; <.001 (.47)	−.162; [−0.33, 0.01]; .09 (.51)

*Notes*: *Pub PHC* Public primary health care, *Pub SHE* Public secondary health care

### Correlates of satisfaction

Organizational supports (i.e. managerial support, salary and benefits, job security, performance evaluation, reward system, career opportunity and resources availability), physical working conditions, internal social supports, and job characteristics had direct and strong relations with satisfaction in both public and private doctors. Additionally, health-professional politics and external social supports had considerably strong inverse and direct influence respectively on job satisfaction in public doctors. Relations of organizational supports and internal social supports to job satisfaction were nearly 1.5-times stronger in the private than in the public sector (Table 4).

### Correlates of turnover intention

Organizational supports and physical working conditions were negatively related to turnover intention in both sectors. While the strength of relations between organizational supports and turnover intention was nearly identical in both sectors, which for physical working conditions was nearly double in the private than in the public sector. Health-professional politics was found to have a strong positive relation with turnover intension only in the public doctors. Internal social supports and job characteristics had statistically significant negative correlations with turnover intention only in private doctors (Table 4).

### Correlates of burnout

Organizational supports had a strong and inverse influence on burnout in public doctors only. Oppositely, physical working conditions showed a marginally significant indirect relation with burnout in private doctors. Health-professional politics and workloads had a significant and direct influence on burnout only in the public doctors. In contrast, internal and external social supports showed a significant negative influence on burnout only in the private doctors with a fairly stronger relation with external social supports. Job characteristics had a significant negative correlation with burnout in both the public and private doctors with nearly twice stronger in the latter (Table 4).

### Multiple regression: Key predictors of work design outcomes

In the public doctors, organizational supports were found the strongest predictor of all outcomes and explained nearly 60% of a total predicted variance in satisfaction, which in case of turnover intention and burnout were 88 and 68% respectively. Notably, the variance in satisfaction and turnover intention in the private doctors was predicted merely by organizational supports, while that in burnout was explained by external social supports. Table 5 presents the values of *R*, *R* ^2^ changes, standardized betas, and *F* changes including *p*-values of the key predictors of outcomes in both the sectors.

**Table 5:** Multiple regression statistics of key predictors of outcomes of interest in the public and private doctors

Model *Provider type*	*R*	*R* ^*2*^	Standardized β (*p*-value)	Change statistics			
				*R* ^*2*^ change	*F* change	df 1 / 2	Sig. *F* change
A. Model: satisfaction							
*Public doctors*							
1. Organizational supports	.371	.137	.19 (.006)	.137	36.32	1 / 228	<.001
2. Job Characteristics	.439	.193	.23 (<.001)	.056	15.55	1 / 227	<.001
3. Working condition	.469	.220	.17 (.009)	.027	7.90	1 / 226	.005
4. Health-professional politics	.484	.235	- .13 (.040)	.015	4.28	1 / 225	.040
*Private doctors*							
1. Organizational supports	.539	.290	.539 (<.001)	.290	42.91	1 / 105	<.001
B. Model: turnover intention							
*Public doctors*							
1. Organizational supports	.416	.173	−.37 (<.001)	.173	47.85	1 / 228	<.001
2. Health-professional politics	.443	.196	.16 (.012)	.023	6.36	1 / 227	.012
*Private doctors*							
1. Organizational supports	.351	.123	−.35(<.001)	.123	15.02	1 / 107	<.001
C. Model: burnout							
*Public doctors*							
1. Organizational supports	.209	.044	−.16 (.019)	.044	10.41	1 / 228	.001
2. Health-professional politics	.255	.065	.15 (.023)	.021	5.23	1 / 227	.023
*Private doctors*							
1. External social supports	.291	.085	−.292 (.002)	.085	9.91	1 / 107	.002

*Note*: Sig. indicates significance

## Discussion

This study aimed to identify the relationships of work characteristics to job satisfaction, turnover intention, and burnout among doctors of the public primary, public secondary, and private facilities of the district health system of Bangladesh and whether or not the relations differ among those target groups, and to identify the major predictors of those outcomes of interest in them.

Overall, all significant correlations were quite modest in magnitude; however, since the sample size was adequately large the correlations are meaningful. We found no significant differences between the responses on work characteristics and outcomes of interest in the public primary and secondary level doctors which indicate similar effects of the work characteristics on the public doctors’ job satisfaction, turnover intention and burnout status; however, there are significant differences between the public and private sector doctors. This section will briefly describe those distinctions.

Organizational supports predicted incomparably high variations in satisfaction and turnover intention in both the public and private doctors. However, the predicted variations in burnout were notably low in both sector doctors (Table 5).

### Work characteristics and doctors’ job satisfaction

Empirically, organizational supports such as good managerial support, performance evaluation and feedback, reward systems (promotion, increment), resource availability and career growth opportunity positively influence employees’ satisfaction [24, 25]. A qualitative study of Bagheri et al. [10] at Tabriz, Iran also found that structural and managerial factors had potential influence on job satisfaction of health care staff. Our study findings are consistent with those studies.

In the public health sector of Bangladesh, senior General Physicians (GPs) usually subjugate the administrative sub-cadre without prior administrative/managerial training and are successively promoted as an ‘Upazilla (sub-district) Health and Family Planning Officer’ (i.e. head of a upazilla primary health care system) and then to Civil Surgeon (i.e. head of a district health system) and so on. At upazilla and district level hospitals, postgraduate doctors have to work under the administrators who most often are GPs. Tensions between public doctors and administrators are common [3]; thus, low organizational and internal social supports are phenomenal.

Low salary, benefits and job security correlate to low satisfaction [8], which likely explains the strong direct correlation between organizational supports and satisfaction in private doctors since their job security, salary, benefits and career growth opportunities are implicitly neglected.

Notably, mean scores of organizational supports were substantially low in both the public and private sectors although lower in the public (Table 3). All these findings indicate the crucial need of improving overall organizational supports to enhance job satisfaction in both the public and private doctors. The study of Goetz et al. at Kenya also reported the similar findings [11].

Further, the study of Campion [26] reported that unpleasant working conditions often are related to low job satisfaction, reflected in favours our findings of significant positive correlations between physical working conditions and satisfaction in both the public and private doctors. Thus, improvement in physical working conditions is necessary to improve doctors’ satisfaction in both sectors.

Investigating health-professional politics as a determinant of work design outcomes is unique since only one study of Andaleeb et al. [17] implicitly addressed the issue that transfer and promotion of public doctors often are influenced by political identity rather than a fair evaluation of performance. Our empirical findings confirm that health- professional politics have negative impacts on job satisfaction but direct relations with turnover intention and burnout only in the public doctors.

The degree of internal social supports (e.g. interpersonal relationships with supervisor and co-workers) is directly correlated with job-holders’ satisfaction (Janssen et al., 1999; Humphrey et al.*,* 2007) [4, 7]. Our study also implies that improvements in the internal social supports would improve doctors satisfaction in both the sectors.

Consistent with the study of Fried & Ferris [27] that suggested that job characteristics (e.g. autonomy, feedback from job, task variety, task significance and skill variety) are positively correlated with job satisfaction, we also suggest that there are needs of improving overall job characteristics to improve job satisfaction of the public doctors.

### Work characteristics and doctors’ turnover intention

There is sufficient evidence of inverse relationships between organizational supports and turnover intention. For example; well-defined career growth reduces turnover intention [28], and conversely, low salary, benefits, and job security correlate with high turnover intention [8]. A study at Ghana reported significant indirect associations of low carrier growth opportunity, inefficient management and organizational commitment to turnover intention [13]. Our findings are consistent with those studies for both the public and private doctors. Inverse correlations between internal social supports and turnover intention were reported by Janssen et al. [7] and Humphrey et al. [4]. These studies support our findings for the private doctors.

In Bangladesh, doctors’ turnover from the private to the public sector is restricted by the Public Service Commission with an age barrier of no later than 32 years. Oppositely, public doctors are free to leave from public to private service, and turnover is common in the private doctors between private sector facilities. There are examples of public specialist doctors’ leaving their jobs for tertiary level private hospitals after completing the minimum service duration criteria for pension. Notably, we found that turnover intentions had wide standard deviations in both the public and private sectors (Table 3). This indicates that there are doctors who work with intentions to leave organization (in the public sector) or frequent changing of facilities (in the private sector). Likely, these are the senior specialist doctors experiencing job dissatisfaction and/or high demands with opportunities of high incentives in the private job markets. This unstable situation is a barrier to optimal output of health sector.

Consistent with the report of Gray-Toft & Anderson [29] and Bonenberger et al. [13], we also noticed significantly large inverse relations between satisfaction and turnover intention in both sector doctors (Table 4). This indicates that improvement of satisfaction is essential to diminish turnover intention in both sector doctors.

### Work characteristics and doctors’ burnout

Because of the doctors’ job nature, they are among those professionals who often experience high ‘job demands’ e.g. workload, inferior physical working conditions, time pressure and emotional demands [30]. Individual incapability of coping with job demands may lead to job strain including burnout [31]; for instance, excess workload often is related to emotional burnout [8]. Further, Bakker et al. [32] reported that favourable ‘job resources’ (e.g. organizational and social supports, and autonomy) hold the potential of buffering the stressor effects of job demands. We found that organizational supports are stronger stressors for burnout in the public doctors than in the private; however, in the latter, social supports buffer burnout more strongly.

Considering the long weekly working hours and daily professional activities, workloads are high in both the public and private doctors. However, we found that workloads are quite weak predictor of burnout in both sectors. Reportedly, over 80% of the public doctors were involved in dual-practice. The common predictor of dual-practice is to supplement low salary in the public sector [3, 33]. In the competitive private market high patient-loads (i.e. workloads) are desirable as directly related to income, which may serve as a buffer against burnout. However, why high workloads are not related to burnout in the private doctors still is not entirely clear.

Overall mean scores of burnout both in the public and private doctors were markedly low (Table 3). Further, our model predicted burnout only weakly. Out of three items of the scale, two items measured burnout indirectly: ‘I have become intolerant to my patient since I started my job’ and ‘I feel a lack of confidence to continue my job effectively and efficiently’. Answers to both scales may possibly be affected by social desirability response bias resulting in low scores. Taking into account the challenges of measuring job stress as described by Bakker & Demerouti [34] in critically appraising the two widely used job stress models namely the ‘demand-control model’ of Karasek, and the ‘effort-reward imbalance model’ of Siegrist, we realize that measurement of burnout in the doctors of the public-private mixed health system of Bangladesh is also a complex issue. A model integrating organizational, social and job characteristics for direct measuring of burnout with possible other stressors and buffering attributes including individual characteristics and background could be suitable for the Bangladeshi context.

### Strengths and limitations

To the best of our knowledge, this is the first study addressing the work design issues on the health system of Bangladesh. Doctors from all divisions of the country responded with a high response rate. It was impossible to use a random sampling method; thus, selection bias cannot be excluded. However, by sampling doctors from both the public and private sectors with a gender distribution similar to the national ratio and with diverse specializations in a unitary health system, we claim that our study results are representative to the key work design issues of the district health system of Bangladesh. The use of a self-administered questionnaire removed the risk of interviewer bias although we cannot ignore the possibility of social desirability response bias on issues relating to professional dignity. We investigated the influence of professional politics on doctors which is an additional issue to other studies of this field. This study would be an essential evidence for policymakers for re-designing the human resource management policy of the health system of Bangladesh as well as of other countries with similar contexts. Further studies are necessary for an in-depth exploration of the opinion of key stakeholders for recommending strategic and sustainable work re-design for the public-private mixed health system of the country.

## Conclusion

Work design in the health system of Bangladesh is in need of substantial development. Inadequate organizational supports (e.g. lack of performance evaluation and reward system, low salary, benefits and career growth opportunities, inefficient managerial supports, scarce resources) as the most potential predictors exert an adverse influence on satisfaction and turnover intention in both sectors as well as on burnout for public doctors; burnout in private doctors has a significant indirect relation with external social supports. The adverse influence of health-professional politics on satisfaction, turnover intention and burnout in the public doctors is alarming. Physical working conditions have strong adverse effects on job satisfaction in doctors of both sectors and on turnover intention only in private doctors. While internal social supports are found as important determinants of doctors’ job satisfaction in both sectors, job characteristics only predicts public doctors’ satisfaction.

Overall, improvement of the doctors’ job satisfaction in both the public and private sectors is crucial through addressing the organizational supports system, which in turn would reduce turnover intention and burnout as well, since all those outcomes are mostly under the potential influence of similar organizational factors. However, social and job characteristics also have meaningful effects on work design outcomes. Improvement of doctors’ overall welfare relating to their job is essential to improve their performance as well as to enhance optimal output of the health system of Bangladesh.

## Additional file

Additional file 1: Study questionnaire. This document illustrates the tool which was used as a self-administered questionnaire for collecting data from the target doctors. Mostly standard items were used with some were adapted. (DOC 156 kb)

## Abbreviations

BMA: Bangladesh Medical Association
GP: General Physician
MANOVA: multivariate analysis of variance
