# Supplementary material for: Relationships of work characteristics to job satisfaction, turnover intention, and burnout among doctors in the district public-private mixed health system of Bangladesh
Source: BMC Health Serv Res. 2017 Jun 20;17:421. doi: 10.1186/s12913-017-2369-y (PMC5480190; doi:10.1186/s12913-017-2369-y)
Supplement: Additional file 1: — Study questionnaire. This document illustrates the tool which was used as a self-administered questionnaire for collecting data from the target doctors. Mostly standard items were used with some were adapted. (DOC 156 kb) [file 12913_2017_2369_MOESM1_ESM.doc]

**Study questionnaire**

**General instructions:**

*Filling out this questionnaire will take approximately 30 minutes. If you cannot complete answering in one attempt, please do not hesitate to do this in several turns. You need to tick/circle only one selected option for each question, which is the most factual you believe by heart.*

**Section 1: P**ersonal and professional background

| **Q. ID** | **Question** | **Response** | **Code** |
| --- | --- | --- | --- |
| 1.1 | What is your date of birth? |  | |
| 1.2 | Gender | - Male | **1** |
|  |  | - Female | **2** |
| 1.3 | Title of your current position | - Medical Officer | **1** |
|  |  | - Junior consultant | **2** |
|  |  | - Senior consultant | **3** |
|  |  | - Residential Medical Officer | **4** |
|  |  | - Other (please specify below)   …………………………………… | **#** |
| 1.4 | What is your qualification/s  (Please write only Bangladesh Medical and Dental Council (BMDC) approved degrees) | - MBBS | **1** |
|  |  | - Diploma | **2** |
|  |  | - FCPS | **3** |
|  |  | - MD | **4** |
|  |  | - MS | **5** |
|  |  | - MPH | **6** |
|  |  | - Other: ……………………… |  |

| **Q. ID** | **Question** | **Response** | **Code** |
| --- | --- | --- | --- |
| 1.5 | What is your specialization?  (Please write down only BMDC recognized specializations in the space to the right) | - General Physician | **1** |
|  |  | - Specialization in:   …………………………………… | **2** |
| 1.6 | Organization where you are working: | - Public sector | **1** |
|  |  | - Private sector | **2** |
| 1.7 | How many years are you working as a doctor? | ………….Years | |
| 1.8 | If you are a public doctor: Where are you posted now? | - Sub-district Health Complex | **1** |
|  |  | - District hospital | **2** |
| 1.9 | Are you doing private practice or working part-time in other private clinics or hospitals? | - Yes | **1** |
|  |  | - No | **2** |
| 1.10 | Please tick your working division | Dhaka / Chittagong / Khulna / Rajshahi / Sylhet / Barisal / Rangpur | **#** |
| 1.11 | Please name your working district |  | |

***Important notes on how to answer the questions / statements in the following sections: All statements / questions are measured on a 5-point rating scale: 1 = strongly disagree, 2 = disagree, 3 = neither agree nor disagree, 4 = agree and 5 = strongly agree. You need to circle/tick only one out of five options that you believe factual.***

**Section 2: Work characteristics domains**

**2.1 Job characteristics**

| **Ques.** | **Statement** | **Respondent’s rating** | | | | |
| --- | --- | --- | --- | --- | --- | --- |
| 2.1.1 | I have adequate freedom to make my duty schedule | **1** | **2** | **3** | **4** | **5** |
| 2.1.2 | I have freedom of choosing methods to treat patients *(such as advising diagnostic tests, drugs and surgical or conservative treatments)* | **1** | **2** | **3** | **4** | **5** |
| 2.1.3 | I do the same tasks repeatedly in everyday practice *(e.g. only outdoor or indoor or emergency duties or operation etc.)* | **1** | **2** | **3** | **4** | **5** |
| 2.1.4 | I have the opportunity to do different types of tasks every-day (e.g. indoor, outdoor, emergency, surgery, patient discharge etc.) | **1** | **2** | **3** | **4** | **5** |
| 2.1.5 | I enjoy doing same tasks repeatedly | **1** | **2** | **3** | **4** | **5** |
| 2.1.6 | I enjoy different tasks in a day | **1** | **2** | **3** | **4** | **5** |
| 2.1.7 | I feel my job has significant effect on people’s lives. | **1** | **2** | **3** | **4** | **5** |
| 2.1.8 | I often get patients’ feedback that conveys recognition of how well I am doing my job. | **1** | **2** | **3** | **4** | **5** |
| 2.1.9 | I need different types of skills to do my tasks | **1** | **2** | **3** | **4** | **5** |

**2.2 Social characteristics (internal and external social supports)**

| **Ques.** | **Statement** | **Respondent’s rating** | | | | |
| --- | --- | --- | --- | --- | --- | --- |
| 2.2.1 | I get adequate support from my supervisor | **1** | **2** | **3** | **4** | **5** |
| 2.2.2 | My colleagues are adequately supportive and friendly | **1** | **2** | **3** | **4** | **5** |
| 2.2.3 | I feel my patients are adequately satisfied. | **1** | **2** | **3** | **4** | **5** |
| 2.2.4 | I have a good relationship with patients. | **1** | **2** | **3** | **4** | **5** |
| 2.2.5 | Most often I experience friendly relationships with and support from people in the community. | **1** | **2** | **3** | **4** | **5** |

**2.3 Organizational characteristics**

| **Organizational supports** | | | | | | |
| --- | --- | --- | --- | --- | --- | --- |
| 2.3.1 | I get adequate managerial supports *(e.g. support from UHFPO / Civil surgeon / superintendent / director or manager of private clinic / hospital)* | **1** | **2** | **3** | **4** | **5** |
| 2.3.2 | There is a transparent system of performance evaluation in my organization. | **1** | **2** | **3** | **4** | **5** |
| 2.3.3 | There is a reward system, such as promotion and increment, which is based on performance evaluation | **1** | **2** | **3** | **4** | **5** |
| 2.3.4 | Salary and benefits are adequate in my organization | **1** | **2** | **3** | **4** | **5** |
| 2.3.5 | There is job security in my organization | **1** | **2** | **3** | **4** | **5** |
| 2.3.6 | There is an opportunity for career development | **1** | **2** | **3** | **4** | **5** |
| 2.3.7 | There is an established performance feedback system from the management level | **1** | **2** | **3** | **4** | **5** |
| 2.3.8 | There are adequate supplies of resources ( e.g. equipment, drugs and diagnostic facilities) | **1** | **2** | **3** | **4** | **5** |

| **Physical working conditions and facilities** | | | | | | |
| --- | --- | --- | --- | --- | --- | --- |
| **Ques.** | **Statement** | **Respondent’s rating** | | | | |
| 2.3.9 | The building and cleanliness are adequate to do my daily tasks | **1** | **2** | **3** | **4** | **5** |
| 2.3.10 | The workplace is free from excessive noise and crowds | **1** | **2** | **3** | **4** | **5** |
| 2.3.11 | The work place is comfortable in terms of temperature and humidity | **1** | **2** | **3** | **4** | **5** |
| 2.3.12 | Home to work-place transport is good | **1** | **2** | **3** | **4** | **5** |
| 2.3.13 | There are adequate living facilities surrounding my work-place | **1** | **2** | **3** | **4** | **5** |
| **Workload** | | | | | | |
| 2.3.14 | The quantity of tasks to complete my daily responsibilities is quite high | **1** | **2** | **3** | **4** | **5** |
| 2.3.15 | To complete daily tasks I need to work hard under time pressure | **1** | **2** | **3** | **4** | **5** |
| 2.3.16 | How many days in a week are you involved in treating patients? |  | | | | |
| 2.3.17 | On an average, daily how many hours do you invest to treat patients in the public and/or private sector/s? | …………hours/day | | | | |
| 2.3.18 | Approximately how many patients do you treat per day? |  | | | | |
| **Health-professional politics** | | | | | | |
| 2.3.18 | Professional political identity often is more important for reward and career development than performance | **1** | **2** | **3** | **4** | **5** |
| 2.3.19 | Professional politics often discourage my commitment to the organization and job | **1** | **2** | **3** | **4** | **5** |
| 2.3.20 | Professional politics often hampers working relationships among colleagues | **1** | **2** | **3** | **4** | **5** |
| 2.3.21 | Professional politics are a barrier to maximize productivity of the public health sector | **1** | **2** | **3** | **4** | **5** |
| 2.3.22 | Professional politics are a barrier to maximize productivity of the private health sector | **1** | **2** | **3** | **4** | **5** |

**Section 3: Work design outcomes**

**3.1 Job satisfaction**

| **Ques.** | **Statement** | **Respondent’s rating** | | | | |
| --- | --- | --- | --- | --- | --- | --- |
| ***Scenarion1: At this stage of your service, you are adequately experienced about the overall situations of your job. Suppose, you need to decide from the beginning about this job and position, what would you decide? Please see the statements below-*** | | | | | | |
| 3.1.1 | I would again accept this job without any hesitation or doubt | **1** | **2** | **3** | **4** | **5** |
| 3.1.2 | I would consider working in another organization (for example: from public to private or from private to public sector or in another hospital) | **1** | **2** | **3** | **4** | **5** |
| 3.1.3 | I would definitely change the present organization | **1** | **2** | **3** | **4** | **5** |
| ***Scenarion2: According to your overall evaluation of the current job, how do you measure or rate that this is the job you wanted in the beginning of your professional career? Please see the statements below-*** | | | | | | |
| 3.1.4 | This job meets most of my expectations | **1** | **2** | **3** | **4** | **5** |
| 3.1.5 | This job meets some of my expectations | **1** | **2** | **3** | **4** | **5** |
| 3.1.6 | This job meets nearly none of my expectations | **1** | **2** | **3** | **4** | **5** |
| ***Scenario 3: If any of your friends or relatives seek your suggestions to work in a similar organization as yours (e.g. Public or private sector), what you will tell him/her? Please see the statements below-*** | | | | | | |
| 3.1.7 | I would strongly recommend my organization | **1** | **2** | **3** | **4** | **5** |
| 3.1.8 | I would be careful and only explain the real situation; so that he / she can decide | **1** | **2** | **3** | **4** | **5** |
| 3.1.9 | I would strongly suggest not to work in my organization | **1** | **2** | **3** | **4** | **5** |

**3.2 Turnover intention**

| **Ques.** | **Statement** | **Respondent’s rating** | | | | |
| --- | --- | --- | --- | --- | --- | --- |
| 3.2.1 | If I have an opportunity I would leave this organization, | **1** | **2** | **3** | **4** | **5** |
| *3.2.2* | *I may leave this organization after maturing the job duration for pension* | ***1*** | ***2*** | ***3*** | ***4*** | ***5*** |
| *3.2.3* | *I shall stay in this organization even if I have an opportunity to leave* | ***1*** | ***2*** | ***3*** | ***4*** | ***5*** |

**3.3: Burnout**

***In this very last part, there are three statements. Each statement has seven options. Please select only one option for each statement that suits you best.***

| **Ques** | **Statement** | **Responses** | **Rating** |
| --- | --- | --- | --- |
| 3.3.1 | I feel burnout from my work (e.g. I feel emotionally unable to face works of another day) | Never | **1** |
|  |  | Once in a year | **2** |
|  |  | Once in a month or less | **3** |
|  |  | A few times a month | **4** |
|  |  | Once a week | **5** |
|  |  | A few times a week | **6** |
|  |  | Every day | **7** |
| 3.3.2 | I have become intolerant to my patients since I started my job | Never | **1** |
|  |  | Once in a year | **2** |
|  |  | Once in a month or less | **3** |
|  |  | A few times a month | **4** |
|  |  | Once a week | **5** |
|  |  | A few times a week | **6** |
|  |  | Every day | **7** |
| 3.3.3 | I feel lack of confidence to continue my job effectively and efficiently' | Never | **1** |
|  |  | Once in a year | **2** |
|  |  | Once in a month or less | **3** |
|  |  | A few times a month | **4** |
|  |  | Once a week | **5** |
|  |  | A few times a week | **6** |
|  |  | Every day | **7** |

**The End**
